# The Disproportionate Increase of the Intraoperative Flexion and Extension Gap Space after Posterior Cruciate Ligament Resection in Total Knee Arthroplasty

**DOI:** 10.3390/jcm10184228

**Published:** 2021-09-17

**Authors:** Kao-Chang Tu, Han-Ting Shih, Shih-Chieh Tang, Cheng-Hung Lee, Wei-Jen Liao, Shun-Ping Wang

**Affiliations:** 1 Department of Orthopaedics, Taichung Veterans General Hospital, Taichung 40705, Taiwan; shark310751@gmail.com (K.-C.T.); h901920@gmail.com (H.-T.S.); tom0857tom0857@gmail.com (S.-C.T.); Lch51015101@gmail.com (C.-H.L.); cavaliarjames@gmail.com (W.-J.L.); 2 Department of Food Science and Technology, Hungkuang University, Taichung 433304, Taiwan; 3 College of Medicine, National Chung Hsing University, Taichung 40227, Taiwan; 4 Sports Recreation and Health Management Continuing Studies-Bachelor’s Degree Completion Program, Tunghai University, Taichung 40704, Taiwan

**Keywords:** flexion gap, gap balancing, posterior cruciate ligament, PCL resection, total knee arthroplasty, tensioner

## Abstract

Purpose: Maintaining gap balance is critical for total knee arthroplasty (TKA). This study aimed to elucidate if the extension–flexion gaps would be changed with posterior cruciate ligament (PCL) intact (PI) and PCL resection (PR) during TKA. The flexion gaps were measured using two methods, open-(Fo) and closed-chain position (Fc), based on the definition of kinetic chain position, respectively. Methods: This retrospective study enrolled a total of 33 patients who underwent posterior-stabilized (PS) TKA for symptomatic advanced osteoarthritis of knees. After bone cuts were completed, the extension–flexion gaps before and after PCL resection during TKA were measured using a calibrated tensioning device set at a 100 Nm distraction force. To further differentiate the effect of thigh weight on the 90° flexion gap, two varied methods of examination, either in closed chain (Fc) or open chain (Fo) were performed. Results: The increases in the 90° knee flexion gap after PCL resection were measured by both methods, i.e., ΔFc (PR-Fc—PI-Fc): 2.04 ± 2.06 mm, *p* < 0.001; and mean ΔFo (PR-Fo—PI-Fo): 1.64 ± 1.36 mm, *p* < 0.001. However, there were no differences between ΔFc and ΔFo before and after PCL resection. A greater amount of flexion gap was identified in open chain than in closed chain after PCL resection, and the PR-Fo and PR-Fc were 14.36 ± 3.13 and 11.40 ± 3.47 (*p* < 0.001), respectively. Conclusions: The resection of PCL during TKA distinctly increased the flexion gap, but not the extension gap. This disproportionate increase of the gap will cause a gap balance mismatch. The tensioning maneuver in open-chain was more effective to detect the gap differences than in closed-chain before and after PCL resection during TKA.

## 1. Introduction

In addition to the aging population and the increase in obesity, younger knee injury patients and the expansion of TKA indications have led to the rapid expansion of total knee arthroplasty in the past decade [1,2]. For the success of TKA, correct alignment, stability, and balanced extension–flexion gaps are essential [3,4]. TKA instability is a common cause (approximately 27%) of revision operation [5]; therefore, maintaining the balance of soft tissue during TKA is crucial.

The gap balancing of TKA, first used by John Insall in 1979, emphasized the establishment of identical flexion and extension spaces before implantation [6]. Cruciate-retaining (CR) and posterior-stabilized (PS) prostheses are the primary types of TKA. The main difference between the two is that the PCL is retained with CR types but sacrificed with PS types. The posterior cruciate ligament (PCL) resection in PS TKA may cause a lax flexion gap that affects the extension–flexion gap mismatch [7,8,9]. Both prostheses have comparable prognoses and high postoperative satisfaction [10]. However, because the cam–spine mechanism in PS TKA cannot completely rebuild the functional capacity of the intact PCL [11,12,13], surgeons should pay attention to whether PCL resection will affect the flexion gap and result in flexion instability. Although it remains unclear whether PCL resection leads to relevant instabilities of the flexion gap, a greater midflexion laxity was found after PS TKA than after a CR procedure [14]. However, evidence is inconsistent regarding whether PCL resection increases the flexion gap. Many studies have stated that PCL resection has no effect on the extension gap but increases the flexion gap by 1.1–1.9 mm [15,16]. Other studies have indicated that PCL resection does not affect the flexion gap in PS TKA or cause a flexion–extension gap mismatch [17,18,19], and that the flexion gap is affected only when PCL resection is combined with medial collateral ligament (MCL) release [20,21]. This discrepancy in the literature on the effect of PCL resection on the extension–flexion gaps may be related to the measurement methods, the recruited research participants (cadaveric or clinical study), or whether PCL resection was combined with MCL release.

The purpose of this study was to identify whether PCL resection affected the extension–flexion gaps when MCL was protected without any release. In addition, considering that the weight of the thigh might affect flexion gap measurement, we employed two methods to measure the flexion gap, namely the open-chain and closed-chain methods. We postulated that the flexion gap would increase after PCL resection and that the flexion gap measured during open-chain flexion would be greater than that measured during closed-chain flexion.

## 2. Materials and Methods

### 2.1. The Enrollment of the Participants

This retrospective study was approved by the Institutional Review Board (No. CE21157B) of our institution. We recruited patients receiving primary PS TKA from August 2020 to May 2021. The inclusion criteria were having end-stage osteoarthritis, namely Kellgren and Lawrence grade 4, and being older than 18 years. The exclusion criteria were secondary osteoarthritis caused by fractures, ligament injuries, or inflammatory arthritis (e.g., the systemic lupus erythematosus, and rheumatoid arthritis); knee ligament operation or osteotomy; and PCL insufficiency or rupture before or during operation.

### 2.2. Surgical Interventions

**Figure 1:**
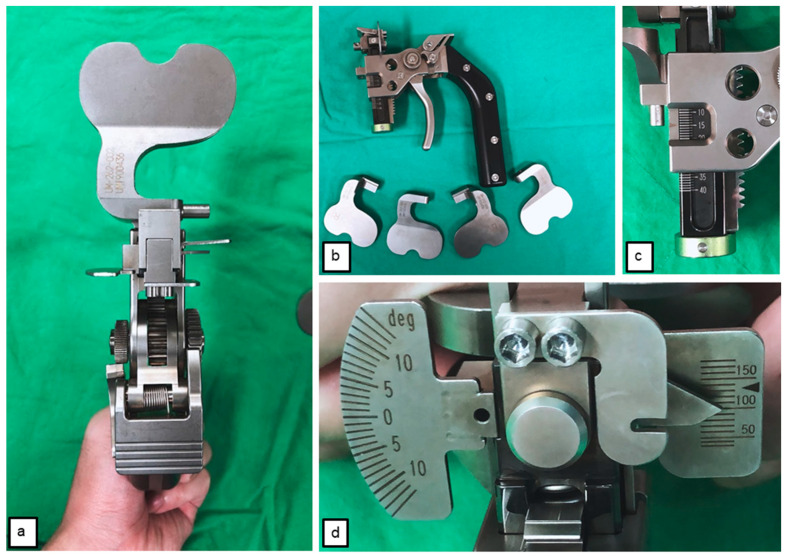
The tensioner: (**a**) top view; (**b**) side view; (**c**) ruler at the side; (**d**) 100 Nm force set to distract the knee joint.

All patients received TKA in our institution by a single senior surgeon in this study. A calibrated tensioner was used for measurement during the operation. The posterior-stabilized (PS) prostheses, U2 Knee (United Orthopedic, Taipei, Taiwan), were implanted with cemented fixation in all participants under anesthesia through a standard middle-line incision with medial parapatellar arthrotomy. The posterior reference was used to finish the bone cut of the femoral condyle. The anterior and posterior femur were completed using a cutting jig and chamfer cuts. A PCL-protecting retractor (i.e., no-touch PCL retractor) was employed, and a 3.0 K pin was inserted in front of the PCL insertion to avoid damage to the PCL during the tibial cut. Subsequently, after the medial and lateral meniscus was removed, the integrity of the PCL was confirmed visually and by manual test. According to Nowakowski’s recommendation, a tension device was used to give a 100 Nm distraction force to simulate the physiological tension of the natural knee to measure the extension–flexion gaps after PCL intact (PI) and PCL resection (PR) [20,22] (Figure 1). When performing the bone cut during TKA and before recording the measurement results, medial and lateral soft tissue release was not implemented in order to avoid errors and maintain consistency in research conditions.

### 2.3. Data Collection of Image Parameters

All patients underwent preoperative and postoperative full-length, standing AP view X-rays, in addition to a standing knee X ray. The hip–knee–ankle angle (HKA), the lower limb coronal alignment defined as the angle between the mechanical axis of the femur and the tibia, was measured in full-length, standing AP view. The measurement of the postoperative tibia slope, defined as the angle between the longitudinal axis of the tibia and tibial implant, was performed in the sagittal plane of the standing knee X-ray [23].

### 2.4. Tensioner Measurements for Extension–Flexion Gaps

**Figure 2:**
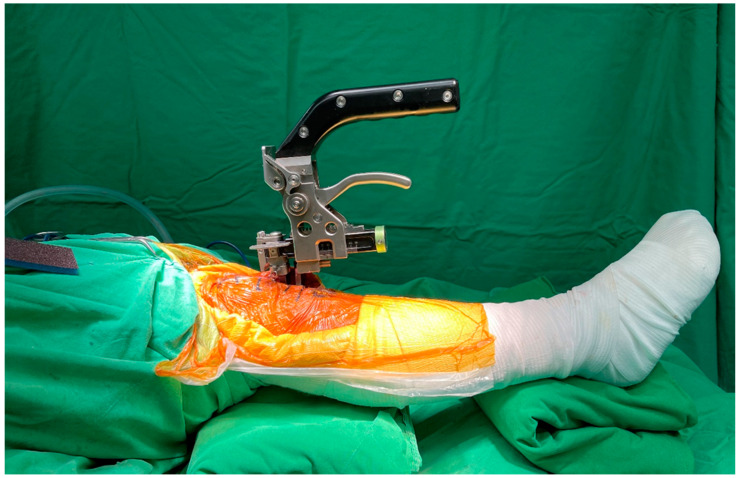
Measurement of the extension gap (E gap) using the tensioner. The lower limb was placed in full extension and the patella was placed in the anatomical position during measurement.

**Figure 3:**
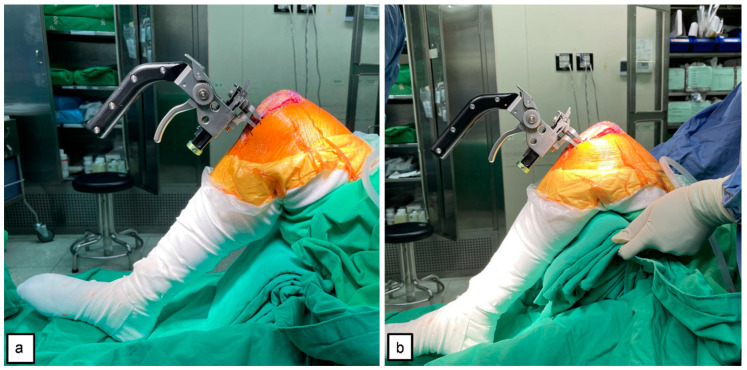
The flexion gap being measured by the tensioner: (**a**) closed-chain flexion gap (Fc gap) of the knee at 90° flexion without thigh support and the foot placed on the operating table; (**b**) open-chain flexion gap (Fo gap) of the knee at 90° flexion with thigh support and the foot suspended off the table.

During the measurement, the tensioner was positioned between the end of the distal femur and tibia plateau, and the patella was placed in the anatomical position. The tensor device, equipped with a calibration spring, was gradually and steadily adjusted from 0 Nm to 100 Nm, the targeted tension set in this study. At this time, the gap distance could be measured and easily read from the ruler on the side of the tensor device (Figure 1c). During extension gap (E gap) measurement, the patient’s lower limb was fully extended (Figure 2), and the flexion gap was measured using two methods: (1) flexion gap under closed chain (Fc gap) (Figure 3a); and (2) flexion gap under open chain (Fo gap) (Figure 3b). Then, the PCL was resected from its femoral insertion. The entirety of the PCL release was verified by visual inspection and manual palpation. After that, we placed the tensioner in the joint space again and repeated the measurement. All the above measures were repeated two times. Notably, between each measure, the tensioner device was released to ensure no residual forces were retained and that no forces were introduced by constraining the knee when changing position. The gap measurements of 10 participants were performed as described above by another surgeon, the data was collected and calculated for interclass correlation, and the intraclass correlation coefficient (ICC) was used to assess the reliability of this tensioner device.

### 2.5. Data Analysis

The continuous variables are presented with means and standard deviations, and the categorical data are presented with frequencies and percentages. A paired *t*-test was used to calculate the comparison of changes before and after PCL resection. Analyses of gap spaces and gap differences between groups were subjected to independent *t*-tests. A correlation analysis was conducted using the Pearson correlation coefficient to validate the intraobserver and interobserver reliability. An independent *t*-test was used for the binary independent variable, and Spearman’s rho coefficient was used for the continuous dependent variable. All statistical analyses were performed using IBM SPSS Statistics for Windows, version 22 (IBM Corp., Armonk, NY, USA). The significance level was *p* < 0.05.

## 3. Results

### 3.1. Demographics and Characteristics of Enrolled Cases

From July 2020 to May 2021, 37 patients underwent primary TKA. After excluding two patients with PCL deficiency, one with traumatic osteoarthritis knee, and one with rheumatoid arthritis, 33 participants were included for analysis. Their demographic data are presented in Table 1.

### 3.2. Gap Space Changes before and after PCL Resection

There was no significant difference in the extension gap changes after PCL resection. A 90° flexion gap was measured in closed- and open-chain flexions, and a significant difference was observed between the gap changes measured using the two methods. In the closed-chain measurement, the flexion gap increased by 2.04 ± 2.06 mm after PCL removal (PI-Fc 9.27 ± 3.05 vs. PR-Fc 11.31 ± 3.44); *p* < 0.001). In the open-chain measurement, the flexion gap increased by 1.64 ± 1.36 mm after PCL removal (PI-Fo: 12.72 ± 3.07 vs. PR-Fo: 14.36 ± 3.13; *p* < 0.001) (Table 2).

**Figure 4:**
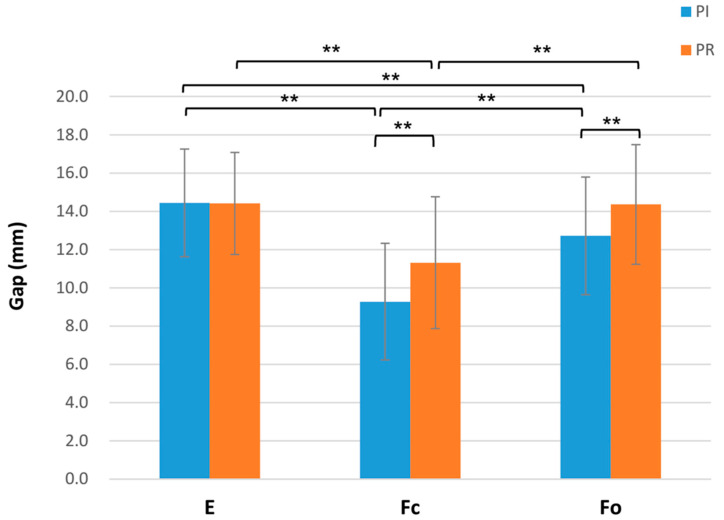
Comparisons of the extension gap (E), flexion gap in closed-chain (Fc), and open-chain (Fo) before and after PCL resection. Paired-t test; ** *p* < 0.01.

Before PCL resection, the PI-E gap (14.44 ± 2.82 mm) was significantly larger than the PI-Fc gap (9.27 ± 3.05 mm, *p* < 0.001) and the PI-Fo gap (12.72 ± 3.07 mm, *p* = 0.003). After PCL resection, the PR-E gap (14.41 ± 2.67 mm) was still significantly larger than the PR-Fc gap (11.31 ± 3.44 mm) (*p* < 0.01) but was not significantly different from the PR-Fo gap (14.36 ± 3.13 mm) (*p* = 0.950). No significant difference in the flexion gap change after PCL resection was observed between the closed-chain and open-chain flexions (ΔFc: 2.04 ± 2.06 mm; ΔFo: 1.64 ± 1.36 mm; *p* = 0.294). However, after the final PCL resection, the flexion gap value measured using open-chain flexion was significantly higher than that measured using closed-chain flexion, by 2.97 ± 1.93 mm (PR-Fo: 14.36 ± 3.13 mm; PR-Fc: 11.40 ± 3.47 mm; *p* < 0.001) (Figure 4).

### 3.3. Factors Correlated to Flexion Gap Changes with PCL Resection

Subgroup analysis revealed that gender, body-mass index, preoperative deformity, and anesthesia method did not affect the gap differences between the two measurement methods, except that closed-chain flexion was affected by the preoperative ASA grades (Table 3). We further measured the preoperative and postoperative HKA angle and postoperative tibial slope. The mean preop HKA, postop HKA, and postop tibial slope were 4.09 ± 7.22, 1.98 ± 3.77, and 86.47 ± 2.98 degrees, respectively. There were no correlations between these parameters and the Δ flexion gap (Table 4).

### 3.4. Intraclass Correlation (ICC) and Interclass Correlation

The reliability of the tensioning device was evaluated using intraclass and interclass correlation. The intraobserver and interobserver ICCs were excellent (ICC > 0.9) for all measurements. All procedures were completed without intraoperative complication.

## 4. Discussion

Our results supported the study hypotheses. When the MCL was retained, the flexion gap, whether measured using closed- or open-chain flexion, significantly increased by an average of 2.04 mm and 1.64 mm after PCL resection, respectively, while the extension gap was not affected. Thigh weight affected the measurement of the 90° knee joint flexion gap: both before and after PCL resection, the open-chain flexion gap was significantly larger than for the closed-chain method. Subgroup analysis revealed that the Δ flexion gap measured in the closed-chain method, but not in open-chain position, was affected by patients’ preoperative ASA grades. At present, we cannot explain why there was such a difference. This may require more attention to clarify its relevance in the future. Furthermore, the measurements conducted using a tensioning device had a high consistency. To the best of our knowledge, this was the first study to compare the effects of PCL resection on the tibiofemoral space measured while preserving the integrity of the MCL. This was also the first study that compared the difference in flexion gaps measured by the open- and closed-chain methods. Our results can serve as a reference for surgeons to assess the gap balance during TKA.

We discovered that PCL resection increased the flexion gap, consistent with previous studies [15,16,19,24,25,26]. Park et al. found that PCL resection with simultaneous extensive MCL release during TKA obviously increased the mean flexion gap, by 3.95 mm [27]. This factor may increase the flexion gap except for the laxity of the medial soft tissue, so their results indicated considerably higher values than ours. Kayani et al. retained MCL integrity and used the open-chain method for measurement. The results showed that PCL resection increased the flexion gap by 1.1 cm [15], which agreed with our results.

The flexion gap is typically measured by placing the thigh on the tibia during TKA, and is thus affected by thigh weight; however, such influence is rarely discussed in the literature. According to the cadaver study conducted by Kaneyama et al. [28], thigh weight loss does affect the flexion gap, and our in vivo study reached the same conclusion.

Other studies have indicated that PCL resection does not affect the flexion gap in PS TKA or cause a flexion–extension gap mismatch [17,18,19]; Burkhart et al. and Oshima et al. reported that MCL release causes flexion gap changes but not PCL resection under a similar distraction force to that used in the current study [20,21]. Unfortunately, the study by Burkhart et al. was a cadaveric study that was different from the soft tissue in vivo, which may explain the difference between the results [20]. In their in vivo study, Oshima et al. proposed results inconsistent with our findings. Differing from this study, they performed a pretest before bone resection and measured the gaps while the patella was laterally displaced, rather than in a neutral position, which may also have affected their results [21]. In addition, the femoral insertion of the PCL had two bundles [29]. Therefore, a complete PCL resection was difficult to determine without performing a bone resection. We supposed that the PCL might have been resected incompletely in their study and may have caused insignificant flexion-gap changes [30].

Gap balance between the extension and flexion gaps is the crucial goal in TKA. The final flexion gap will be partially influenced by the prosthetic design, such as the reference for bone cut at the posterior femoral condyle either anterior or posterior, and the thickness of the posterior condyle of the implant. Since the bone cut reference of the TKA implant we used in this study was a posterior, we cut the fixed bone level at 9 mm from the posterior femur condyle. Unlike the TKA cutting guide designed with anterior reference, the level of bone cut of the TKA prosthesis in this study at the posterior femoral condyle was not influenced by femoral size choosing. According to our measurement, the extension gap and flexion gap using the open-chain method (Fo) seemed identical after PCL release. The balanced gaps during TKA in most of our cases might have been caused by the design of the implant, or the cutting guide using the posterior reference. Identifying this issue of gap balance while considering the prosthesis design being used and the influence of PCL resection on flexion gap should be addressed to prevent gap imbalance.

Our findings clinically provide surgeons with the ability to facilitate intraoperative extension–flexion gaps when balancing and ligament tensioning during PS TKA. The increase in the flexion gap following PCL resection may also help to explain why deep flexion is improved in PS TKA compared with CR TKA, but with relatively high flexion instability [7]. The present study also demonstrated that the flexion-gap differences measured with open-chain flexion were less affected by patients’ ASA grades. The evaluation of the flexion gap with the open-chain method may be a useful way to assess extension–flexion gaps and soft-tissue balancing in TKA clinically and in future research. Follow-up studies should be conducted to examine whether such objective measurement of gaps enhances patients’ reported outcomes, and to determine its influence on long-term clinical survivorship.

### Limitations

This study had several limitations. First, any retrospective research has potential information and selection bias. Second, all participants had end-stage osteoarthritis. Although the medial soft tissue of the study patients was protected during operation, differences in the degrees of deterioration of their soft tissue contributed to interindividual differences. Third, the sample size was relatively small, and most participants were men. Moreover, the amount of the applied force requires further verification. Although we used 100 Nm of distraction force, which is the amount used in many studies, more research is required to confirm whether this is an appropriate amount. Despite these limitations, all assessments were undertaken by a single experienced surgeon using a standardized protocol with a calibrated tensioner that had high agreement in ICCs to reduce the methodological heterogeneity.

## 5. Conclusions

Both open- and closed-chain methods revealed that sacrificing PCL during PS TKA resulted in an increase in flexion gaps even if the medial soft tissue was preserved. The tensioning maneuver in the open-chain method was more effective in detecting the gap differences than in the closed-chain method. Therefore, we recommend assessing the change in the extension–flexion gap ratio after PCL resection using the open-chain method for achieving satisfactory gap balancing.

## Figures and Tables

**Table 1:** Demographics and characteristics of enrolled cases.

Characteristics		
Case numbers		33
Age (years)		69.39 ± 9.34
Gender, *n* (%)		
	Male	20 (60.6%)
	Female	13 (39.4%)
Side, *n* (%)		
	Right	17 (51.5%)
	Left	16 (48.5%)
BMI		27.64 ± 3.57
ASA grade, *n* (%)		
	I	1 (3.0%)
	II	23 (69.7%)
	III	9 (27.3%)
Preoperative HKA, *n* (%)		
	Varus	27 (81.8%)
	Valgus	6 (18.2%)
Preoperative HKA (degrees)		4.09 ± 7.22
Anesthesia, *n* (%)		
	GA	15 (45.5%)
	SA	18 (54.5%)

ASA, American Society of Anaesthesiologists; BMI, body mass index; HKA, hip–knee–ankle angle; GA, general anesthesia; SA, spinal anesthesia. Continuous data are presented as mean ± SD.

**Table 2:** The gap space changes before and after PCL resection.

	Joint Space (mm)	*p* Value
Extension		0.838
PI-E	14.44 ± 2.82	
PR-E	14.41 ± 2.67	
ΔE (PR-E—PI-E)	−0.03 ± 0.8	
90° flexion gap with closed chain (Fc)		<0.001 **
PI-Fc	9.27 ± 3.05	
PR-Fc	11.31 ± 3.44	
ΔFc (PR-Fc—PI-Fc)	2.04 ± 2.06	
90° flexion gap with open chain (Fo)		<0.001 **
PI-Fo	12.72 ± 3.07	
PR-Fo	14.36 ± 3.13	
ΔFo (PR-Fo—PI-Fo)	1.64 ± 1.36	

Note: paired-*t* test; ** *p* < 0.01. PI: PCL intact; PR: PCL resection. Continuous data presented as mean ± SD.

**Table 3:** The factors correlated to flexion gap differences measured by two methods.

		ΔFc (mm) (Mean ± SD)	*p* Value	ΔFo (mm) (Mean ± SD)	*p* Value
Gender			0.274		0.406
	male	2.36 ± 2.25		1.80 ± 1.38	
	female	1.55 ± 1.68		1.38 ± 1.35	
BMI			0.920		0.289
	<25	2.09 ± 1.21		1.19 ± 1.14	
	≥25	2.02 ± 2.28		1.79 ± 1.42	
ASA grade, *n* (%)			0.038 *		0.138
	I–II	2.49 ± 2.11		1.42 ± 1.37	
	III	0.84 ± 1.38		2.22 ± 1.21	
Preoperative deformity			0.361		0.326
	Varus	2.20 ± 2.05		1.76 ± 1.30	
	Valgus	1.33 ± 2.10		1.14 ± 1.64	
Anesthesia			0.521		0.125
	GA	2.30 ± 2.24		2.04 ± 1.39	
	SA	1.83 ± 1.93		1.29 ± 1.28	

Note: BMI, body mass index; ASA, American Society of Anaesthesiologists; GA, general anesthesia; SA, spinal anesthesia; Independent *t*-Test; * *p* < 0.05.

**Table 4:** Coefficient of correlation with Δ flexion gap after PCL.

	ΔFc	ΔFo
Age	0.060	0.232
BMI	−0.249	0.163
Pre-op. HKA	0.166	0.165
Post-op. HKA	0.179	0.245
Post-op. tibia slope	0.094	0.104

Note: Spearman’s rho coefficient; BMI, body mass index; HKA, hip–knee–ankle angle.

## Data Availability

The data that support the findings of this study are available from the corresponding author upon reasonable request.
